# Effect of intrauterine insemination treatment on sexual function and quality of life for infertile women

**DOI:** 10.12669/pjms.344.14789

**Published:** 2018

**Authors:** Emre Sinan Gungor, Olcay Seval, Gulşah Ilhan, Fatma Ferda Verit

**Affiliations:** 1 Emre Sinan Gungor, M.D. Suleymaniye Maternity Research and Training Hospital, Department of Obstetrics and Gynecology, Istanbul, Turkey; 2 Olcay Seval, M.D. Suleymaniye Maternity Research and Training Hospital, Department of Obstetrics and Gynecology, Istanbul, Turkey; 3 Gulşah Ilhan, M.D. Suleymaniye Maternity Research and Training Hospital, Department of Obstetrics and Gynecology, Istanbul, Turkey; 4 Prof. Dr. Fatma Ferda Verit, Suleymaniye Maternity Research and Training Hospital, Department of Obstetrics and Gynecology, Istanbul, Turkey

**Keywords:** Intrauterine insemination, Infertile women, Sexual function, Quality of life, depression

## Abstract

**Objectives:**

To evaluate the effect of intrauterine insemination (IUI) on sexual functioning, quality of life and psychological well-being.

**Methods:**

One hundred and thirty four infertile women going to IUI treatment as study group and 134 women who do not report any infertility complaint attending to gynecology clinic for routine control as control group were enrolled. Demographic data of the patients were collected. Patients were asked to complete Female Sexual Functioning Index (FSFI), Beck Depression Inventory (BDI) and SF 36 form.

**Results:**

Total FSFI score (mean±SD) for study group was 23.4±4.1 and 24.8±3.4 for control group (p<0.05). This means a lower sexual function for patients going to IUI. There were also statistically significant differences according to subscales of FSFI scores for sexual desire, arousal and satisfaction. Mean±SD scores for Beck Depression Inventory analysis was 18.6±9.8 for study group and 18.5±7.1 for control group. According to SF-36 scores, there were statistically significant differences between the groups for four subscales: Role physical,bodily pain,general health and vitality.

**Conclusions:**

Going to an IUI treatment has negative effects on FSFI scores and some of SF 36 scores but we did not find a significant negative effect on BDI scores.

## INTRODUCTION

Infertility is a disease characterized by the failure to establish a clinical pregnancy after 12 months of regular, unprotected sexual intercourse.^1^ About 15% of the worldwide population is infertile and this condition expected to rise.^2^

Some infertile couples have some psychological difficulties, including; lack of marital satisfaction, impairment of relationships, lack of sexual satisfaction, forced timing of intercourse, loss of confidence in relation to sex, decreased libido and negative emotional effects.^3^-^5^ There are many studies investigating the impact of infertility on female sexual function. Some of them reported higher prevalence of sexual dysfunction in infertile females,^6^,^7^ whereas some others revealed no significant difference between fertile and infertile females.^8^,^9^

Infertility may also have a negative effect on the Quality Of Life (QOL) for the woman and maybe a cause of developing depression. QOL is defined as ‘an individual’s perception of their position in life in the context of the culture and value systems in which they live and concerns incorporating physical health, psychological state, social relations and personal beliefs.^10^ SF-36 is one of the best well defined test for assessing measure of self-reported health.

Depression may be another problem which a female may experience if she has infertility. In the literature, there are some articles concluding that infertility may cause depression and some articles deny such a relation.^11^-^13^

Intra Uterine İnsemination (IUI) is a procedure performed at earlier stages of infertility treatment compared to İnvitro Fertilization (IVF) and IVF is considered to be the end point treatment stage for infertility by the patients. So when compared with IVF procedure, starting an IUI cycle may be less stressful for the couple. To our knowladge, there is no study invastigating the sexual problems and quality of life of females who are going to IUI treatment.

The aim of our study was to evaluate the effect of starting an IUI cycle on sexual functioning, quality of life and psychological well-being for females in comparison with a group of presumed fertile women.

## METHODS

Women aged between 20-40 years attending to infertility clinic of Suleymaniye Maternity Research and Training Hospital for IUI treatment were included in this prospective study as study group (Group 1) between January 2016 and February 2017. Women attending the gynecology clinic for general control without any complaints were taken as control group (Group 2). A total of 268 patients (134 study group and 134 control group) entered the study. One hundred forty two patients were invited to answer the questionnaries for the study group but six of them refused to fill the FSFI questionnarie and two of them answerred the questionnaries but there were missing data; so these eight women were excluded from the study. One hundred forty five patients were invited to answer the questionnaries for the control group but six patients did not accept to fill the questionnaries and five patients’ demographic data were missing. Non-pregnant and non-lactating, in a sexual relationship during the last 4 weeks were prospectively included in the study. Women with known psychiatric diseases, using antipsychotic drugs, genitourinary infection, genital tract abnormality, physical disabilities, and having chronic medical conditions associated with sexual dysfunction were excluded. Females with partners that had erectile or ejaculation disturbances associated with sexual dysfunction were also excluded.

All participants from the control group had at least one living child. None of the patients in the control group did not have any complaint while attending to hospital. They were just attending to gynaecology clinic for routine control. Infertile women included all those who were experiencing primary or secondary infertility. Participants who had never been able to conceive were diagnosed as primary infertility, whereas those with a previous history of pregnancy were diagnosed as secondary infertility.

Ethical committee approval was taken before starting the data collection. All participants gave informed consent after which demographic data and reproductive history of the patients were collected. Patients were asked to complete three self-administered questionnaires. Study group patients filled the questionnaries for the month just before starting to use the drugs related the IUI practice. All patients underwent stimulated IUI cycles, but administration of hormonal treatment is not a confounding variable because all our patients answered the questionnaries before starting any treatment. For the control group, patients were requested to answer the questionnaries after their physical examination evaluating the previous month. three self-administrated questionnaires were as follows:

1. Short Form Health Survey SF-36: The SF-36 is a 36-item self-administered instrument that measures health status. It consists of eight subscales including Physical Functioning (PF), Role- Physical (RP) (limitations, difficulties due to one’s physical problems), Bodily Pain (BP) (magnitude of pain), General Health (GH), Vitality (VT) (state of being strong and active), Social Functioning (SF), Role-Emotional (RE) (role limitations due to emotional problems) and Mental Health (MH).These eight subscales are scored from 0 to100 where 100 indicates the highest level of health status.^14^ SF 36 was adapted and validated for the Turkish population by Pınar et al.^15^ SF36 can also be scored as two summary measures: Mental Component Summary (MCS) and Physical Component Summary (PCS). PF, RP, BP, GH are evaluated in PCS; and VT, SF, RE and MH are evaluated in MCS.
2. The Female Sexual Functioning Index (FSFI) is a 19-item multidimensional survey measuring six domains including sexual desire, arousal (both subjective and physiological), lubrication, orgasm, satisfaction, and pain. Each domain was scored on a scale of 0 to 6, with higher scores indicating better sexual function for each domain.^16^ It was validated for Turkish population and was used to assess female sexual function among women.^17^ The individual domain scores were totaled and multiplied by a predetermined factor to weigh each domain equally.^16^ A score ≤26.5 was defined as female sexual dysfunction.^18^
3. Beck Depression Inventory (BDI) was used to assess depression for the study and control groups. It consisted of 21 questions on particular aspects of depression related symptoms. Total score ranged from 0 to 63. A score of ≥17 was chosen as clinically significant symptoms of depression.^18^ Turkish version of the BDI has been tested and validated in the Turkish population.^19^

### Statistical Analyses

Mean, standard deviation, median, minimum, maximum value frequency and percentage were used for descriptive statistics. The distribution of variables was checked with kolmogorov-simirnov test. Mann-whitney U test was used for the comparison of quantitative data. Chi-Square test was used for the comparison of the qualitative data. SPSS 22.0 was used for statistical analyses. p<0.05 was considered as statistically significant.

## RESULTS

The mean age was 30.1±4.5 for study group and 29.5±4.5 for control group. Also there was not a statistically significant difference for BMI rates between the groups (26.2±3.6 vs 25.5±3.1 respectively for study and control group). 97/134 (72.3%) of patients were primary infertile in study group. Mean duration of infertility was 19.3±4.6 months for study group. All patients in the control group had at least one child. Demographic characteristics of the patients are shown in Table-I.

**Table-I:** Demographic characteristics of the patients.

		Study group		Control group		p

		Mean±s.d.	Median	Mean±s.d.	Median
Age in years (mean±SD)		30.1±4.5	30.0	29.5±4.5	29.0	0.167
Body Mass Index (kg/m2)		26.2±3.6	26.0	25.5±3.1	25.2	0.148
Gravidy		0.1±0.3	0.0	1.0±0.8	1.0	0.000
Parity		0.3±0.5	0.0	1.4±0.9	1.0	0.000
Educational Status	Primary School	83±61.5%		67±49.6%		0.057
	High School	45±33.3%		52±38.5%		
	University	7±5.2%		16±11.9%		
Family status	Nuclear	131±97.0%		130±96.3%		0.735
	Extended	4±3.0%		5±3.7%		
Occupational status	Employee	36±26.7%		43±31.9%		0.349
	Housewife	99±73.3%		92±68.1%		
Marital Status	Married	135±100.0%		135±100.0%		-
	Single	0±0.0%		0±0.0%	
Use of antidepressants		15±11.1%		12±8.9%		0.543
Cigarette smoking		40±29.6%		36±26.7%		0.588
Use of alcohol		13±9.6%		11±8.1%		0.669
Doing exercise		19±14.1%		31±23.0%		0.060
s.d. Standard Deviation						

Forty two out of 134 patients (31.3%) had an FSFI score more than 26,5 and 49/134 (36.5%)of our control group had an FSFI score more than 26.5. But when we analysed the overall FSFI score (mean±SD) for study group was 23.4±4.1 and 24.8±3.4 for control group (p<0.05). This means a lower sexual function in infertile women. There were also statistically significant differences between groups according to subscale of FSFI scores for sexual desire (3.6±1 vs 4.5±0.9, respectively p<0.05), arousal (4.1±1.1 vs 4.6±1, respectively, p<0.05) and satisfaction (3.7±1 vs 4.1±1, respectively, p<0.05). There were no statistically significant differences for other subscales of FSFI between groups.

The mean±SD scores for Beck Depression Inventory analysis was 18.6±9.8 for study group and 18.5±7.1 for control group and there was not a statistically significant diffrence. Twenty out of 134 (14.8%) of infertile patients experienced severe depression and 12/134 (8.9%) of control group had severe depression and this was not statistically significant too.

When the groups were analysed according to SF-36 scores, there were statistically significant differences between the groups for four subscales: Role physical (63,5±28 vs 71.9±26.9 respectively, p<0.05), bodily pain (28.4±20.3 vs 63.8±23 respectiveley, p<0.05), general health (51.6±12 vs 56.9±16.4 respectively, p<0.05) and vitality (50.3±11.7 vs 55.4±13 respectively, p<0.05). There were no statistically significant differences for other subscales of SF-36 between the groups. Scores for all patients and for all questionnaries are shown in Table-II.

**Table-II:** Mean BDI, FSFI, SF 36 scores of study and control group.

	Study Group		Control Group		p

	Mean±s.d.	Median	Mean±s.d.	Median
Beck Depression Score	18.6±9.8	18.0	18.5±7.1	18.0	0.798
Normal	64±47.4%		57±42.2%		
Mild depression	51±37.8%		66±48.9%		
Severe depression	20±14.8%		12±8.9%		
***FSFI***					
Total FSFI	23.4±4.1	23.6	24.8±3.4	24,9	0.001
Desire	3.6±1.0	3.6	4.5±0.9	4,2	0.000
Arousal	4.1±1.1	4.2	4.6±1.0	4,8	0.000
Lubrication	4.1±1.0	4.2	4.1±0.9	3,9	0.215
Orgasm	3.8±1.2	4.0	3.9±0.9	4,0	0.641
Satisfaction	3.7±1.0	3.6	4.1±1.0	4.0	0.015
Dyspareunia	4.1±1.0	4.0	4.3±4.6	3.6	0.096
***SF-36***					
Physical function	66.1±19.6	70.0	65.5±18.4	65.0	0.728
Role Physical	63.5±28.0	75.0	71.9±26.9	750	0.018
Bodily pain	58.4±20.3	57.5	63.8±23.0	67.5	0.017
General health	51.6±12.0	50.0	56.9±16.4	55.0	0.002
Vitality	50.3±11.7	50.0	55.4±130	55.0	0.000
Social functioning	60.2±19.4	62.5	58.1±21.9	50.0	0.340
Role emotional	50.4±33.1	66.7	53.1±29.2	66.7	0.531
Mental health	53.5±12.7	52.0	56.6±15.2	56.0	0.109

BDI: Beck Depression Inventory, FSFI: Female Sexual Functioning Index, SF-36: Short Form Health Survey.

There was a positive correlation between total FSFI scores and SF36 physical function score. There was also a positive correlation between FSFI dyspareunia score and SF 36 physical function scores (p<0.05). We could not find any other correlation between FSFI sores and SF 36 scores (Table-III).

**Table-III:** Correlation between FSFI and SF 36 scores.

		Physical function	Role Physical	Bodily Pain	General health	Vitality	Social functioning	Role emotional	Mental health
Total FSFI	r	0.162	0.036	0.041	0.062	-0.011	-0.055	-0.052	0.065
	p	0.008	0.555	0.502	0.311	0.857	0.366	0.395	0.288

Desire	r	-0.047	0.065	0.002	0.085	0.077	-0.011	0.020	0.000
	p	0.442	0.287	0.969	0.163	0.209	0.855	0.741	0.999

Arousal	r	0.079	0.100	0.031	0.079	-0.017	0.039	0.038	0.052
	p	0.197	0.101	0.615	0.195	0.781	0.519	0.538	0.390

Lubrication	r	0.077	-0.089	-0.055	0.041	-0.084	-0.091	-0.138	0.085
	p	0.206	0.143	0.366	0.504	0.166	0.137	0.052	0.163

Orgasm	r	-0.050	-0.043	-0.058	-0.055	-0.025	-0.073	-0.059	-0.090
	p	0.413	0.480	0.339	0.368	0.683	0.232	0.330	0.140

Satisfaction	r	0.116	-0.043	0.101	0.077	0.078	-0.015	-0.048	0.074
	p	0.057	0.478	0.098	0.207	0.199	0.808	0.431	0.228

Dyspareunia	r	0.225	0.066	0.081	-0.030	0.050	-0.009	-0.044	0.116
	p	0.000	0.279	0.185	0.618	0.412	0.877	0.469	0.057

## DISCUSSION

Sexual function is one of the important components of health and overall quality of life. Infertility has a major negative impact on women’s and also couples quality of life and sexual life. It is associated with feelings of loss of control, diminished self-esteem, anxiety and depression.^7^

As we found lower FSFI scores for infertile patients, similar to us Oddens at al and Drosdzol et al reported lower sexual life satisfaction for infertile patients when compared with healthy controls.^11^,^20^ Oddens et al found significant difference for desire and satisfaction subgroups; Drosdzol et al found difference for arousal, orgasm and pain subgroups. We found significant difference between groups for sexual desire, arousal and satisfaction subgroups of FSFI. Also Millheiser at al and Hentschel et al reported lower FSFI scores for sexual desire and arousal subdomains.^6^,^21^ From these results it may be concluded that , sexual desire and arousal are the most problematic subdomains in infertile patients. Howbeit problems in satisfaction subgroup is revealed to be common in Turkish women.^17^

Prior studies have indicated that infertile women report more depressive symptoms than controls. In a study of 338 infertile women and 39 healthy controls, women who had a duration of infertility between two to three years reported a higher prevelance of depression compared with the control group.^22^ In a study conducted in the Netherlands, 281 women seeking infertility treatment reported higher rates of depressive symptoms compared to the 289 healthy controls.^11^ However, another recent study concluded that depression is uncommon in women seeking infertility treatment.^12^ Although it may be anticipated that infertility may cause depression, like Brasile et al we also could not find different BDI scores between infertile patients and control group. The relationship between female infertility and depression remains somewhat unclear. As 99% of people living in our country, Turkey, is Muslim; our population may accept infertility as a destiny. As a result of this acceptance, although infertility affects sexual function and some of SF 36 scores, its effect on BDI scores is limited.

Drosdzol et al and Souter et al reported lower QoL among infertile women but different from us Souter et al reported lower scores in all SF-36 categories as we found lower scores for role physical, bodily pain, general health and vitality subgroups.^20^,^23^ These results are comparable to ours. We could find limited correlation between FSFI and SF 36 scores. This correlation was only on physical function subdomain.

Although İstanbul is the biggest and most well-known city of Turkey, our hospital’s patients are generally from lower income group and come from eastern citys of Turkey. Different from Western cultures where marriages are thought to be result of love, for such a population like ours, marriage is based on family constitution. Therefore low marital satisfaction especially among women may be a common problem. Higher BDI scores, lower quality of life and higher frequencies of sexual problems may be more common for both our infertiile and also fertile group of patients.

### Limitations of the study

The sample size might be larger to generalize the findings. Another thing we did not evaluate was the husband’s sexual function index. The etiology of infertility in these couples was not assessed.

Our SF 36 analyzes also shows similar results that generally physical component of the questionnarie is negatively effected for infertile patients (role physical, bodily pain and general health). Only vitality subgroup of SF 36 questionnarie which is related with mental component summary is worser in infetile patients. The second finding of our study is that there are no group differences in symptoms of depression, is a good news that adds to existing evidence that, although infertility is distressing, psychopathology is no more common among infertile than fertile women. Infertile patients may perceive IVF treatment as the last chance of having a baby and a more stressful process but our study demonstrated that planning an IUI treatment also have unfavourable effects. Although there are several studies reporting investigating the QOL and sexual problems of infertile women going to IVF or ICSI treatment,^24^ to our knowladge it is the first study to date investigating FSD and quality of life for infertilite women going to IUI treatment.

## CONCLUSIONS

We investigated the effect of going to an IUI treatment on sexual functioning and quality of life on infertile women and found lower FSFI and SF 36 scores but we did not find a significant negative effect on BDI scores. Worse sexual functioning and quality of life probably indicates that anticipating intrauterine insemination treatment is a stressful life event. We may help them by normalizing their feelings and by explaining that it is common to feel less interested in sex leading up to treatment and make sure that they may seek help from a counsellor if this persists.

### Authors’ Contribution

**ESG:** Conceived, designed and editing of manuscript.

**OS, GI:** Did data collection and manuscript writing.

**FFV:** Did review and final approval of manuscript.
